# Cost-effectiveness analysis of integrated community case management delivery models utilizing drug sellers and community health workers for treatment of under-five febrile cases of malaria, pneumonia, diarrhoea in rural Uganda

**DOI:** 10.1186/s12936-021-03944-3

**Published:** 2021-10-18

**Authors:** Patrick Lubogo, John Edward Lukyamuzi, Deo Kyambadde, Alex Aboda Komakech, Freddy Eric Kitutu, Edgar Mugema Mulogo

**Affiliations:** 1 Pharmacy Department, Mbarara University of Science and Technology (MUST), Mbarara, Uganda; 2 Pharmacy Department, Makerere University College of Health Sciences, Kampala, Uganda; 3 School of Public Health, Makerere University College of Health Sciences, Kampala, Uganda; 4 Department of Community Health, Mbarara University of Science and Technology (MUST), Mbarara, Uganda

**Keywords:** Drug sellers, Community health workers (CHWs), Integrated community case management (iCCM)

## Abstract

**Background:**

Malaria, pneumonia and diarrhoea continue to be the leading causes of death in children under the age of five years (U5) in Uganda. To combat these febrile illnesses, integrated community case management (iCCM) delivery models utilizing community health workers (CHWs) or drug sellers have been implemented. The purpose of this study is to compare the cost-effectiveness of delivering iCCM interventions via drug sellers versus CHWs in rural Uganda.

**Methods:**

This study was a cost-effectiveness analysis to compare the iCCM delivery model utilizing drug sellers against the model using CHWs. The effect measure was the number of appropriately treated U5 children, and data on effectiveness came from a quasi-experimental study in Southwestern Uganda and the inSCALE cross-sectional household survey in eight districts of mid-Western Uganda. The iCCM interventions were costed using the micro-costing (ingredients) approach, with costs expressed in US dollars. Cost and effect data were linked together using a decision tree model and analysed using the Amua modelling software.

**Results:**

The costs per 100 treated U5 children were US$591.20 and US$298.42 for the iCCM trained-drug seller and iCCM trained-CHW models, respectively, with 30 and 21 appropriately treated children in the iCCM trained-drug seller and iCCM trained-CHW models. When the drug seller arm (intervention) was compared to the CHW arm (control), an incremental effect of 9 per 100 appropriately treated U5 children was observed, as well as an incremental cost of US$292.78 per 100 appropriately treated children, resulting in an incremental cost-effectiveness ratio (ICER) of US$33.86 per appropriately treated U5 patient.

**Conclusion:**

Since both models were cost-effective compared to the do-nothing option, the iCCM trained-drug seller model could complement the iCCM trained-CHW intervention as a strategy to increase access to quality treatment.

**Supplementary Information:**

The online version contains supplementary material available at 10.1186/s12936-021-03944-3.

## Background

Internationally, 5.2 million children under the age of 5 years are estimated to have died in 2019. Sub-Saharan Africa has the world’s highest under-5 mortality rate: 76 deaths per 1000 live births. This equates to 1 child in every 13 dying before reaching the age of 5, which is 20 times higher than the rate of 1 in 264 in the region of Australia and New Zealand. In Uganda, the rate of under-5 child mortality is about 46 deaths per 1,000 live births, or one child in every 22 dying before reaching the age of 5 years [1]. This death rate remains higher than the Sustainable Development Goal (SDG) target of fewer than 25 deaths per 1000 live births by 2030.

On a global scale, pneumonia, diarrhoea and malaria remain among the leading causes of death in children under the age of five years as of 2016, accounting for 16, 8 and 5% child mortality rates, respectively [2]. In Uganda, the situation is no different, with malaria, pneumonia and diarrhoea remaining the top three causes of death among children under five years, accounting for about 45–60% of these deaths [3].

In 2010, the Ugandan Ministry of Health launched the community health worker (CHW)-delivered the integrated community case management (iCCM) programme to tackle malaria, pneumonia and diarrhoea among the under-five (U5) children. However, the majority of these U5s in rural Uganda have not fully harnessed the programme’s benefits due to limitations that include inadequate supervision, unreliable medicine, equipment supply chains, low motivation, and retention of CHWs [4, 5]. As a result, many parents in rural Uganda continue to seek U5 febrile care from private drug shops operated by poorly or untrained drug sellers instead of utilizing CHWs trained in iCCM; this propelled various scholars to assess the effectiveness of delivering iCCM intervention via private drug shops [6, 7]. Despite research studies showing the effectiveness of these two delivery models in improving timely appropriate management of malaria, pneumonia and diarrhoea among U5s [6, 8–11], no cost-effectiveness studies have been conducted in Uganda to compare the costs and consequences of utilizing these iCCM delivery models.

This study aims to compare the cost-effectiveness of delivering iCCM intervention through drug sellers against utilizing CHWs in rural areas of Uganda. The study’s findings will contribute to the decision-making process regarding the best delivery model of iCCM intervention based on the costs and outcomes in terms of the number of appropriately treated febrile U5 children, i.e., is the intervention best delivered via CHWs or drug sellers?

## Methods

### Study design

The study was a cost-effectiveness analysis to compare the iCCM delivery model utilizing drug sellers against the model using CHWs. A decision tree analytic model was employed in the economic analysis to construct and structure decisions.

Effectiveness data were obtained from a quasi-experimental study conducted in Southwestern Uganda and the inSCALE cross-sectional household survey that was carried out in mid-Western Uganda. Details of these studies are described elsewhere [6, 12]. In brief: the quasi-experimental study conducted in the South West evaluated the impact of the iCCM intervention on drug seller paediatric fever management and adherence to iCCM guidelines between June 2013 to September 2015. In the intervention arm, 212 care-seeker exit interviews were conducted before, and 285 after; in the comparison arm, 216 care-seeker exit interviews were conducted before, and 268 at the end of the study period [6].

For the inSCALE study, iCCM implementation among CHWs was supported in eight districts in mid-Western Uganda by the Malaria Consortium. Between May and August 2011, a cross-sectional survey was conducted in each of the eight participating districts to assess the impact of the iCCM intervention [12]. Unit costs for items were obtained from the International Drug Price Indicator Guide (2014 edition), iCCM product selection UNICEF guide (2016 edition), Bugoye iCCM project budget, the iCCM costing report for Senegal, and other costing studies [13–15]. International Drug Price Indicator Guide and iCCM product selection UNICEF guide contain international reference prices in US dollars, for essential medicines and medical supplies from pharmaceutical suppliers, international development organizations and government agencies. Other costs were derived from studies conducted in Uganda, close to the Ugandan setting. The micro-costing (ingredients) approach was used to cost the iCCM interventions with the aid of the Community Health Planning and Costing Tool [16]. Cost and effect data were linked using a decision tree model and analysis was carried out with Amua modelling software [17].

### Intervention

The iCCM intervention involved training health workers in the assessment, testing, classification, and treatment of malaria, pneumonia and diarrhoea among children under the age of 5 years. Health care workers were taught how to detect malaria using RDT, how to diagnose pneumonia using respiratory timers, and how to diagnose diarrhoea based on the number of loose stools per day.

In the ICCM delivery model utilizing CHWs, the national iCCM trainer trained health workers (mainly staff from health centres II and III) and district health office staff, as trainers of trainees (TOTs) in a six-day facilitator programme. The TOTs then spent six days training CHWs chosen by their communities. Two trainers were assigned a class of between 25 and 30 CHWs.

Two CHWswere trained in the treatment of malaria, pneumonia and diarrhoea in each village. Free of charge, the trained CHWs received iCCM kits containing rapid diagnostic tests (RDTs) for malaria, respiratory timers, and supplies of artemether/lumefantrine (20 mg/120 mg tablets), amoxicillin (125 mg dispersible tablets), low osmolarity ORS, zinc (20 mg tablets), and rectal artesunate (50 mg). Additionally, the CHWs received a job aid that included the iCCM diagnosis and treatment algorithms, as well as a register for documenting the nature and frequency of all VHT-related activities. Additionally, stock cards, medicine boxes, gloves, cotton wool, and methylated spirit were provided. CHWs were rewarded with raincoats, umbrellas, gumboots, solar lights, and hoes [18].

The iCCM trained-drug seller delivery model consisted of four different components [6]:

1. Selection and training of drug sellers by the study manager, field supervisor, district drug inspector, and district health educator;
2. Provision of materials for information, education and communication (IEC);
3. Subsidized distribution of prepackaged medicines (ACT, amoxicillin dispersible tablets (DT), and zinc sulfate/ORS) to pharmacies. Diagnostics (malaria RDT and respiratory rate counters) and other supplies, such as patient registers, referral slips, supply order forms, and treatment algorithms, were provided free of charge to drug sellers;
4. Monthly support supervision provided by a field supervisor who is either a pharmacist or a clinical physician, accompanied by the district drug inspector and district health educator on occasion.

To improve the community care-seeking practices that affect U5 child health, CHWs delivered messages on fever, care-seeking, diagnostic testing, and treatment adherence, through community meetings, workshops, radio talk shows, announcements, and word-of-mouth [18].

### Estimation of effect

The effectiveness measure used was ‘U5 case of febrile illness appropriately diagnosed and treated’. The phrase refers to any confirmed malaria, diarrhoea or suspected pneumonia case among U5s that received treatment according to treatment guidelines, or any child without malaria, diarrhoea or suspected pneumonia that was not prescribed the recommended drugs to treat malaria, diarrhoea or pneumonia.

Appropriate treatment of uncomplicated malaria entails testing a child with fever or a history of fever with RDT and administering the appropriate ACT regimen if positive. An afebrile child is not tested or prescribed ACT. Children who tested positive for mRDT were given artemether/lumefantrine 20/120 mg DT as follows: 6 tablets for children aged 4–35 months (one tablet twice daily for 3 days), 12 tablets for children aged 36–59 months (two tablets twice daily for 3 days).

A child with a cough and rapid breathing (checked using a respiratory timer to be ≥ 60 breaths per minute (bpm) for a child 0–7 days, ≥ 50 bpm for a child 2–11 months, and ≥ 40 bpm for a child 1–5 years) received an appropriate amoxicillin DT regimen. Amoxicillin DT was not prescribed to a child with a cough and normal breathing. Children with a cough and rapid breathing were given amoxicillin DT 125 mg as follows: 20 tablets for children aged 2–11 months (two tablets twice daily for 5 days); 30 tablets for children aged 12–59 months (three tablets twice daily for 5 days) [6].

Appropriate treatment for non-bloody diarrhoea included administering zinc 20 mg DT and ORS sachets to a child (3 or more loose stools with no blood seen in 24 h). Zinc sulfate was administered as follows: 5 tablets for children aged 2–6 months (half tablet once daily for 10 days), 10 tablets for children aged 7–59 months (one tablet once a day for 10 days). Each child was given two sachets of ORS, and the drug seller or CHW demonstrated how to reconstitute them to the caregiver. Each child was to drink at least half a 300-ml cup of water following each loose stool [6].

Appropriate diagnosis and treatment of U5 febrile illnesses serve as a proxy indicator of the potential for child mortality associated with these illnesses. Malaria cases can rapidly progress to complications and death if treatment is not initiated within the first 24–48 h of symptom onset [19]. Prompt treatment with a complete course of effective antibiotics is critical for pneumonia mortality reduction [20]. ORS and zinc are both effective interventions for reducing diarrhoea-related mortality [21].

The effectiveness data of the drug seller-based delivery model was determined through a quasi-experimental study conducted in Southwestern Uganda. This study is detailed elsewhere [6]. In thisstudy, the iCCM intervention utilizing drug sellers increased the appropriate treatment of uncomplicated malaria, pneumonia symptoms and non-bloody diarrhoea by 80.2% (95% CI 53.2–107.2), 65.5% (95% CI 51.6–79.4), and 31.4% (95% CI 1.6–61.2), respectively.

Effectiveness data for the CHW-based delivery model were obtained from the inSCALE cross-sectional household survey conducted in eight districts of mid-western Uganda. The Malaria Consortium supported the iCCM project through a grant from the Canadian International Development Agency (CIDA). The study's details are available elsewhere [22]. According to Soremekun et al., the coverage rates of appropriate treatment increased 80.4, 51, and 21.5% for uncomplicated confirmed malaria, suspected pneumonia, and non-bloody diarrhoea, respectively.

### Estimation of costs

Economic costing was done from the societal perspective using a micro-costing approach (ingredients approach). The societal perspective is broader than the health care or government perspective and allows comparison with previous studies. Costs that were considered in the societal perspective included health sector costs, costs borne by CHWs or drug sellers, and household costs. It was essential to consider household costs because they can be significant and may deter caregivers from utilizing the iCCM service and cause poverty [23].

Data on the cost of medicines and other health supplies were collected from the International Drug Price Indicator Guide (2014 edition) and iCCM product selection UNICEF guide (2016 edition). Costs for implementation of the iCCM programme were estimated based on the budget of the iCCM project in the Bugoye sub-county and the iCCM costing report for Senegal. Household costs were obtained from costing studies [13–15]. Overhead costs were distributed among the three illnesses based on the prevailing burden of malaria, pneumonia and diarrhoea within the community.

All costs were adjusted to the 2018 price level. For tradable resources, costs in Ugandan shillings (UGX) were exchanged to US dollars (US$) and inflated with US inflation rates. In contrast, the costs of non-tradable resources in UGX were inflated using local inflation rates and then exchanged to US$ [24]. The inflation rates were calculated based on the World Bank GDP implicit price deflators between 2010 and 2018 [25]. The average exchange was UGX3,727 = US$1 by the end of 2018 [26].

### Health sector costs

The total costs of resources for training drug sellers or CHWs, supervision, community sensitization, and management of the iCCM programmes utilizing drug sellers or CHWs were considered health sector costs because the government would need to fund these activities if any of the intervention was to be implemented at scale. The health sector costs mentioned above were estimated based on the budget for the iCCM project being implemented in the Bugoye sub-county in the Kasese district, and the iCCM costing report in Senegal [27]. The training of trainers/supervisors and initial training of drug sellers or CHWs, including the first three months of close support supervision, were considered capital items since their useful lifespan lasted more than one calendar year. These capital costs were annualized at a real discount rate of 3% assuming a useful lifespan of 5 years (annualization factor of 4.58) [28]. The less intense routine supervision and management costs incurred on an annual basis were considered recurrent costs. Costs for community sensitization were similar in both delivery models, thus eliminated.

Medicines, including artemether-lumefantrine, rectal artesunate, amoxicillin, ORS, and zinc, were supplied free of charge to CHWs, and at subsidized prices (mark-up of 50–80%) for drug sellers, and hence also considered health sector costs. Costs for medicines were obtained from the International Drug Price Indicator Guide (2014 edition). A recommended 10% was added to supplier price to cater for shipping cost (freight and insurance) [29] and the wastage rate was assumed to be 10% [30].

Other health sector costs covered mRDT, respiratory timers and health supplies such as stock cards, iCCM registers, examination gloves, cotton wool, and methylated spirit. All these items were supplied free to both drug sellers and CHWs. The prices for these previously mentioned items were obtained by the UNICEF supply catalogue (2016 edition) with 10% added to cater for freight costs [31].

In addition to these items, CHWs were also given medicine boxes, safety boxes, and incentives including raincoats, umbrellas, gumboots, solar lights, and hoes, which were valued at prevailing local prices.

### Costs borne by drug sellers or CHWs

Drug sellers and CHWs invested time in the iCCM programme with no formal pay. Drug sellers or CHWs spent around 30 min per consultation. The median time per household visit was also estimated to be 30 min for CHWs. It was assumed that drug sellers did not perform household follow-up visits for their clients. According to Kasteng et al., the opportunity cost attached to this volunteer time was estimated to be around US$0.34/h based on the median wage rate of alternative work for volunteering communities [32]. Drug sellers received subsidized pre-packaged drugs (ACT, amoxicillin, ORS, zinc), which were sold at a mark-up of 50–80% [7]. Other out-of-pocket costs incurred by CHWs that were not reimbursed included travel expenditure and mobile phone airtime expenditure of about US$2.4 and US$1.5 per annum, respectively [12].

### Household costs for caregivers

Household costs incurred when visiting a drug seller or CHW were divided into direct medical, direct non-medical and indirect costs. Direct medical costs included drug costs or diagnostic costs, while costs such as transport to provider and costs for special food for a febrile child on treatment were considered as direct non-medical costs. Indirect costs related to productivity losses and referred to time spent travelling to a drug seller or CHW and time spent at or with the provider.

Concerning receiving care from CHWs, caregivers travelled an average of 20 min. The waiting times were generally low with caregivers spending only 30 min receiving care from CHWs; the overall time was about 50 [14]. It was assumed that caregivers who sought care from drug sellers spent about 30 min travelling to the provider and another 30 min receiving care from the drug seller [12]. The opportunity cost attached to the caregiver’s time was estimated to be around US$0.34/hour based on the median wage rate within the community [32]. The household costs were obtained from previously conducted household surveys [12, 14], with assumptions being made on the opportunity cost attached to the time the caregiver spent travelling and receiving care from the drug seller.

### Cost-effectiveness analysis

**Fig. 1:**
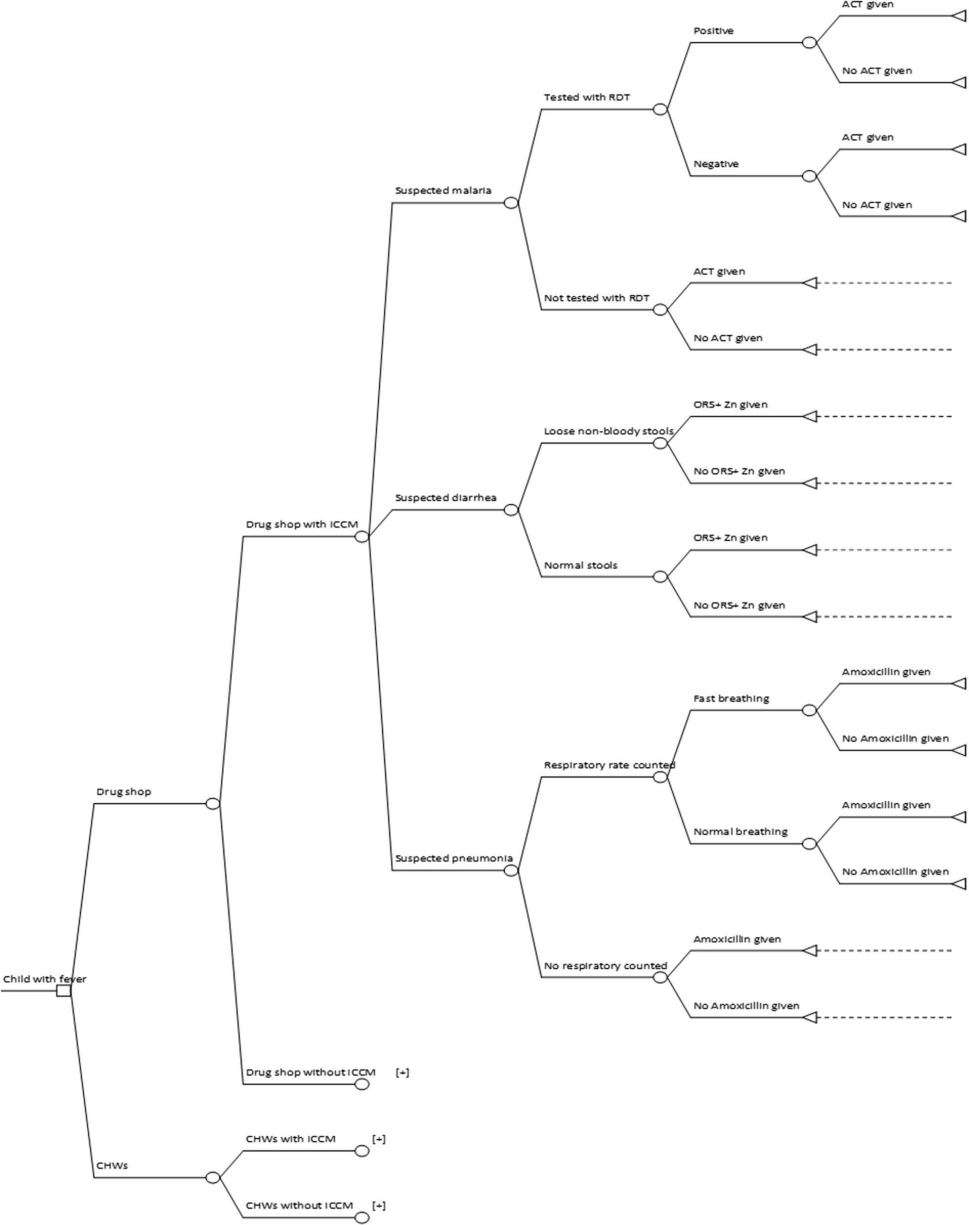
Decision tree modeling delivery of iCCM. [+] sign indicates that the branch uses the same structure as the drug shop with iCCM

Data on the costs and effects were linked through a decision analytical approach [33]. A decision tree was used to model delivery of the iCCM intervention via drug sellers or CHWs as shown in Fig. 1. The probabilities corresponding to the individual tree branches (chance nodes) were obtained from iCCM intervention studies conducted by [6, 7, 12, 15, 34] and are shown in Additional file 1. At the end of each decision tree branch, the treatment of a child with suspected malaria, pneumonia or diarrhoea was classified as appropriate or not.

Societal costs for each decision tree branch were calculated by populating the decision tree with corresponding costs per drug seller or CHW, assuming each saw about 100 febrile U5 cases per year. The overhead costs, such as programme costs, were divided among the three illnesses based on the annual prevalence rate of malaria, pneumonia and diarrhoea for 2017/2018 [3]. The total societal costs and the number of appropriately treated U5 children were calculated by letting 100 children pass through the populated decision tree for each arm. The incremental cost was obtained by subtracting the total cost of the control (CHW) arm from that of the intervention (drug seller) arm. Incremental effect measured as the number of appropriately treated U5s was obtained by subtracting the total effect in the control arm from the total effect in the intervention arm. The incremental cost-effectiveness ratio (ICER) was then calculated as a ratio of the incremental cost (numerator) to the incremental effect (denominator). Therefore, ICER measured the extra cost per additional appropriately treated child when a drug seller is utilized to deliver iCCM instead of a CHW.

The results of this study were presented to guide policymakers. Based on the cost-effectiveness plane, the following classifications were used to explore in which circumstances it might be appropriate to support the drug seller-iCCM delivery model with public funds:

1. iCCM trained-drug seller model dominates: iCCM trained-drug seller intervention is less costly and more effective.
2. iCCM trained-drug seller model is more costly and more effective.
3. iCCM trained-drug seller model is less costly and less effective.
4. iCCM trained-drug seller model has been dominated: iCCM trained-drug seller intervention is more costly and less effective.

From a policymaker’s perspective, if the iCCM trained-drug seller delivery model dominated, it would justify the support of this strategy with public funds. However, this would not mean that the iCCM delivery model utilizing CHWs should be discontinued. The iCCM trained-drug seller model aims to complement the iCCM trained-CHW intervention, and not to substitute it, as a strategy to increase access to quality treatment.

If the iCCM trained-drug seller model were more costly and more effective, the decision on financing it or not would depend on the government’s willingness-to-pay (WTP), as there is no threshold to suggest how much extra money is reasonable to pay per additional case appropriately treated.

If the iCCM trained-drug seller model was less costly but less effective, it is not likely to be considered worthwhile using public funds unless the difference in effectiveness is minimal. In case the iCCM trained-drug seller model was dominated, this would suggest that the intervention should not be supported.

### Sensitivity analysis

One-way sensitivity analyses were conducted by varying the values of individual parameters and assumptions in the decision models to observe the effect on the ICER. The parameters assessed included the probability of seeking care from an iCCM trained-drug seller or CHW, drug seller or CHW utilization of RDTs and respiratory rate timers, malaria, and pneumonia positivity rate.

Scenario analysis was performed for iCCM costs (50% increase or decrease), iCCM effectiveness (50% increase or decrease), and varying prevalence of malaria, pneumonia, and diarrhoea, to assess their impact on the ICER. Probabilistic sensitivity analysis was performed to assess the sensitivity of the ICER to simultaneous variation in the relevant model input parameters by defining probability distributions to selected parameters rather than point estimates [33]. Beta distributions were used for all branch probabilities, except the prevalence of malaria, pneumonia and diarrhoea that were assumed point estimates. Cost parameters were entered in the analysis as point estimates since there was insufficient data to derive their distribution [30]. Simultaneous selection of values from these parameter distributions and point estimates, followed by calculation of ICERs, was performed 10,000 times in Amua modelling software [17] to propagate uncertainty in the ICERs. Uncertainty surrounding the ICERs was summarized by plotting a cost-effectiveness acceptability curve (CEAC), which shows the probability that the use of iCCM delivery models to treat malaria, pneumonia and diarrhoea is cost-effective for different levels of a health policymaker’s hypothetical WTP for an appropriately treated U5 child.

## Results

Health sector unit costs for both iCCM delivery models are summarized in Table 1. The total programme unit cost for the iCCM trained-drug seller delivery model was US$733.33, while the one for the iCCM trained-CHW model was US$692.06. The unit costs for medicines and equipment in the iCCM trained-drug seller intervention amounted to US$2.52 and US$97.69, respectively. The iCCM trained-CHW delivery model had medicine and equipment unit costs of US$3.47 and US$289.63, respectively.

**Table 1:** Health sector unit costs under iCCM strategy in US$ in 2018 and their sources

	iCCM trained-Drug seller	iCCM trained-CHW	Source
Program costs per drug seller or CHW per year			
Training trainers/ supervisors	5.89	4.34	iCCM budget of Bugoye
Training drug sellers or CHWs	25.78	15.81	iCCM budget of Bugoye
Quarterly meetings^a^	0	37.56	iCCM budget of Bugoye
Field supervision visits^b^	161.04	32.16	iCCM budget of Bugoye
Management salaries	540.62	602.19	iCCM budget of Bugoye
Total	733.33	692.06	
Drug and diagnostics costs			
Artemether + Lumefantrine (20 + 120 mg) 6 × 1 blister pack	0.47	0.73	[29]
Artemether + Lumefantrine (20 + 120 mg) 6 × 2 blister pack	0.93	1.43	[29]
First response mRDT) per test	0.78	0.78	[29]
Amoxicillin 125 mg DT per strip of 10 tablets	0.13	0.2	[29]
ORS powder sachet for 1000 ml	0.08	0.13	[29]
Zinc sulphate (20 mg) DT per strip of 10 tablets	0.13	0.2	[29]
Total	2.52	3.47	
Equipment costs			
Respiratory timer	3.84	3.84	[31]
iCCM register	0.59	0.59	[31]
Safety box	12.77	12.77	[31]
Medicines box	0	27.60	[31]
Other supplies^c^	80.49	80.49	iCCM budget of Bugoye
Incentives^d^	0	57.65	iCCM budget of Bugoye
Total	97.67	182.94	

^a^Cost for quarterly meetings and refresher training attended by CHWs at the health facility

^b^Field supervision visits were monthly for the iCCM-drug seller model and quarterly for the iCCM-CHW model

^c^Other supplies include gloves, cotton wool, methylated spirit, and stock cards

^d^Incentives include raincoats, umbrellas, gumboots, solar lights, and hoes

Costs borne by a drug seller or CHW and household costs under the iCCM strategy are summarized in Table 2*.* Unit cost borne by a drug seller under the iCCM strategy was US$1.72, while a CHW bore a unit cost of US$4.47. A U5 caregiver seeking care from an iCCM-trained drug seller incurred a household cost of US$2.8, while a caregiver visiting an iCCM-trained CHW for U5 care incurred a household cost of US$0.59.

**Table 2:** Costs borne by drug sellers or CHWs and household costs under iCCM strategy in US$ in 2018 and their sources

	iCCM trained-Drug seller	iCCM trained-CHW	Source
Cost borne by drug sellers or CHWs			
Value of time lost per consultation^a^	0.17	0.17	[32]
Value of time lost per household visit^b^	0.00	0.17	[32]
Value of time per health facility visit^c^	0.00	0.23	[32]
Non-reimbursed direct travel cost	0.00	2.40	[12]
Non-reimbursed telephone airtime cost	0.00	1.50	[12]
Artemether + Lumefantrine (20 + 120 mg) 6 × 1 pack	0.26	0.00	[29]
Artemether + Lumefantrine (20 + 120 mg) 6 × 2 pack	0.50	0.00	
Artesunate 50 mg rectal capsules	0.60	0.00	
Amoxicillin 125 mg DT per strip of 10 tablets	0.07	0.00	
ORS powder sachet for 1000 ml	0.05	0.00	
Zinc sulphate (20 mg) DT per strip of 10 tablets	0.07	0.00	
Total	1.72	4.47	
Household costs per U5 care-giver			
Direct medical costs^d^	0.2–2.4	0.00	[12]
Direct non-medical cost^e^	0.94	0.31	[12]
Time lost traveling to a health provider	0.17	0.11	[12, 15]
Time spent at a health provider	0.17	0.17	[12, 32]
Total	2.68	0.59	

^a^Refers to the value of time a drug seller or CHW spends diagnosing and treating a sick U5

^b^Refers to the value of time a CHW spends following up on a previously treated U5 case

^c^Refers to the value of time to collect new drugs, deliver service statistics, meet with the supervisor

^d^Direct medical costs include costs for drugs or diagnostic tests

^e^Direct non-medical costs include costs for transport to & from the provider and special food to improve health

Costs and effects are presented for a standard population of 100 U5 children visiting either a drug seller or CHW. The total costs for diagnosing and treating 100 U5 cases of malaria, pneumonia and diarrhoea by a drug seller were US$1,266.35, US$384.18, and US$356.07, respectively (Table 3). For the iCCM trained-CHW model, the total costs for the treatment of 100 U5 cases of malaria, pneumonia and diarrhoea were US$1,132.74, US$336.98, and US$281.58, respectively, as indicated in Table 4.

**Table 3:** Cost of diagnosing and treating malaria, diarrhea, and suspected pneumonia cases per drug seller for 100 children from the societal perspective under iCCM strategy in rural Uganda in 2018 (US$)

	iCCM trained-Drug seller			Drug seller not trained in iCCM		
	Malaria	Pneumonia	Diarrhea	Malaria	Pneumonia	Diarrhea
Health sector costs						
Training trainers/supervisors	3.67	1.40	0.82	0.00	0.00	0.00
Training drug sellers	16.08	6.15	3.56	0.00	0.00	0.00
Quarterly meetings^a^	0.00	0.00	0.00	0.00	0.00	0.00
Field supervision visits^b^	100.44	38.38	22.22	0.00	0.00	0.00
Management salaries	337.19	128.88	74.55	0.00	0.00	0.00
mRDT	234.00	0.00	0.00	0.00	0.00	0.00
Respiratory timers	0.00	3.84	0.00	0.00	0.00	0.00
Drugs	156.59	15.60	40.95	0.00	0.00	0.00
iCCM register	4.42	1.69	0.98	0.00	0.00	0.00
Medicine box	0.00	0.00	0.00	0.00	0.00	0.00
Safety box	7.96	3.05	1.76	0.00	0.00	0.00
Other supplies^c^	50.20	19.19	11.10	0.00	0.00	0.00
Incentives^d^	0.00	0.00	0.00	0.00	0.00	0.00
Total	910.55	218.18	155.94	0.00	0.00	0.00
Costs borne by drug sellers						
Consultation time^e^	17.00	17.00	17.00	12.00	12.00	12.00
Household visit^f^	0.00	0.00	0.00	0.00	0.00	0.00
Health facility visit^g^	0.00	0.00	0.00	0.00	0.00	0.00
Direct travel cost	0.00	0.00	0.00	0.00	0.00	0.00
Airtime cost^h^	0.00	0.00	0.00	0.00	0.00	0.00
mRDT	0.00	0.00	0.00	234.00	0.00	0.00
Drugs	84.32	8.40	22.05	240.90	24.00	63.00
Total	101.32	25.40	39.05	486.9	36.00	75.00
Household costs						
Direct medical costs	126.48	12.60	33.08	478.35	36.00	94.50
Direct non-medical cost^i^	94.00	94.00	94.00	94.00	94.00	94.00
Time lost traveling to health provider	17.00	17.00	17.00	17.00	17.00	17.00
Time spent at health provider	17.00	17.00	17.00	12.00	12.00	12.00
Total	254.48	140.60	161.08	601.35	159.00	217.50

^a^Cost for quarterly meetings and refresher training attended by CHWs at the health facility

^b^Field supervision visits were monthly for the iCCM-drug seller model and quarterly for the iCCM-CHW model

^c^Other supplies include gloves, cotton wool, methylated spirit, and stock cards

^d^Incentives include raincoats, umbrellas, gumboots, solar lights, and hoes

^e^Refers to the value of time a drug seller or CHW spends diagnosing and treating a sick U5

^f^Refers to the value of time a CHW spends following up on a previously treated U5 case

^g^Refers to the value of time to collect new drugs, deliver service statistics, meet with the supervisor

^h^Non-reimbursed travel and airtime costs

^i^Direct non-medical costs include costs for transport to & from the provider and special food to improve health

**Table 4:** Cost of diagnosing and treating malaria, diarrhea, and suspected pneumonia cases per CHW for 100 children from the societal perspective under the iCCM strategy in rural Uganda in 2018 (US$)

	iCCM trained-CHW			CHW not trained in iCCM		
	Malaria	Pneumonia	Diarrhea	Malaria	Pneumonia	Diarrhea
Health sector costs						
Training trainers/supervisors	2.71	1.03	0.60	0.00	0.00	0.00
Training CHWs	9.86	3.77	2.18	0.00	0.00	0.00
Quarterly meetings^a^	23.43	8.95	5.18	0.00	0.00	0.00
Field supervision visits^b^	20.06	7.67	4.43	0.00	0.00	0.00
Management salaries	375.59	143.56	83.04	0.00	0.00	0.00
mRDT	234.00	0.00	0.00	0.00	0.00	0.00
Respiratory timer	0.00	3.84	0.00	0.00	0.00	0.00
Drugs	240.90	24.00	63.00	0.00	0.00	0.00
iCCM register	4.42	1.69	0.98	0.00	0.00	0.00
Medicine box	17.21	6.58	3.81	0.00	0.00	0.00
Safety box	7.96	3.05	1.76	0.00	0.00	0.00
Other supplies^c^	50.20	19.19	11.10	0.00	0.00	0.00
Incentives^d^	35.96	13.73	7.96	0.00	0.00	0.00
Total	1,022.30	237.06	184.04	0.00	0.00	0.00
Costs borne by CHWs						
Consultation time^e^	17.00	17.00	17.00	0.00	0.00	0.00
Household visit^f^	17.00	17.00	17.00	0.00	0.00	0.00
Health facility visit^g^	14.00	5.00	3.00	0.00	0.00	0.00
Direct travel cost^h^	1.50	0.57	0.33	0.00	0.00	0.00
Airtime cost^h^	0.94	0.35	0.21	0.00	0.00	0.00
Drugs	0.00	0.00	0.00	0.00	0.00	0.00
Total	50.44	39.92	37.54	0.00	0.00	0.00
Household costs						
Direct medical costs	0.00	0.00	0.00	0.00	0.00	0.00
Direct non-medical cost^i^	31.00	31.00	31.00	0.00	0.00	0.00
Time lost traveling to health provider	12.00	12.00	12.00	0.00	0.00	0.00
Time spent at health provider	17.00	17.00	17.00	0.00	0.00	0.00
Total	60.00	60.00	60.00	0.00	0.00	0.00

^a^Cost for quarterly meetings and refresher training attended by CHWs at the health facility

^b^Field supervision visits were monthly for the iCCM-drug seller model and quarterly for the iCCM-CHW model

^c^Other supplies include gloves, cotton wool, methylated spirit, and stock cards

^d^Incentives include raincoats, umbrellas, gumboots, solar lights, and hoes

^e^Refers to the value of time a drug seller or CHW spends diagnosing and treating a sick U5

^f^Refers to the value of time a CHW spends following up on a previously treated U5 case

^g^Refers to the value of time to collect new drugs, deliver service statistics, meet with the supervisor

^h^Non-reimbursed travel and airtime costs

^i^Direct non-medical costs include costs for transport to & from the provider and special food to improve health

A cohort of 100 U5 children was passed through the decision-analytic tree. The probabilities associated with the events of the decision tree chance nodes are shown in Additional file 1. In the drug seller arm, the use of an iCCM trained-drug seller to diagnose and treat uncomplicated malaria, pneumonia and non-bloody diarrhoea increased the number of appropriately treated U5 children to 30 per 100. The extra cost from a societal perspective in the iCCM trained-drug seller arm compared to the non-trained drug seller arm was US$591.20 per 100 children leading to an average cost-effectiveness ratio of US$19.86. In other words, using an iCCM-trained drug seller in contrast to a non-trained drug seller would cost US$19.86 extra per appropriately treated U5 child.

For the CHW arm, the use of an iCCM trained-CHW to manage uncomplicated malaria, pneumonia and diarrhoea resulted in an increase in the number of appropriately treated U5 children of 21 per 100 compared to non-trained CHW. The additional cost from a societal perspective in the iCCM trained-CHW arm compared to the non-trained CHW arm was US$298.42 per 100 U5 children leading to an average cost-effectiveness ratio of US$14.13 extra per appropriately treated U5 child. Comparison of the drug seller arm (intervention) with the CHW arm (control) resulted in an incremental effect in the number of appropriately treated U5 children of 9 per 100, and an incremental cost of US$292.78 per 100 appropriately treated children, leading to an incremental cost-effectiveness ratio of US$33.86 per appropriately treated U5 patient, as shown in Table 5.

**Table 5:** Costs, effects, and cost-effectiveness for diagnosing and treating both malaria, pneumonia, and diarrhea per 100 eligible children, as payoffs from the decision-analytic tree model

Variable	Drug seller arm	CHW arm
Number of eligible children for treatment	100	100
Number of appropriately treated cases	30	21
Cost per 100 children (appropriately treated case)	591.20	298.42
Average cost-effectiveness ratio (per appropriately treated case	19.86	14.13
Incremental costs per 100 children (appropriately treated case)	292.78	
Incremental effect per 100 children (appropriately treated case)	9	
Incremental cost-effectiveness ratio (per appropriately treated case)	33.86	

Health sector cost constituted a much larger share of the total societal cost at 64 and 82% in the drug seller and CHW arms, respectively, and it was higher in the CHW arm. The distribution of costs in the iCCM trained-drug seller model was similar to that in the iCCM trained-CHW model, with the management salaries being the most significant cost component. The opportunity cost of the drug seller’s time and CHW’s time comprised 3 and 7% of the total societal cost, respectively. The direct medical cost was the most crucial household cost component in the drug seller arm while the direct non-medical cost was the most significant household cost component in the CHW arm.

The one-way sensitivity analyses explored how varying specific values influenced the ICER from a societal perspective for the drug seller arm against the CHW arm. The ICER was robust to changes in most parameters tested with a few exceptions (Table 6). Decreasing the probability of seeking care from iCCM trained-drug seller to 40% resulted in an ICER that was three-fold higher than the central estimate, making treatment of uncomplicated malaria, pneumonia and non-bloody diarrhoea by iCCM-trained drug seller less attractive from a cost-effectiveness perspective. However, increasing the probability of seeking care from iCCM trained-CHW to 60% resulted in the iCCM trained-CHW delivery model dominating the iCCM trained-drug seller intervention. Decreasing utilization of mRDT by drug sellers to 60% resulted in a four-fold increase in the ICER, making iCCM-trained drug seller intervention a less cost-effective option.

**Table 6:** Sensitivity to selected parameters of the incremental cost-effectiveness ratio (ICER) of iCCM trained-drug seller delivery model vs iCCM trained-CHW in rural Uganda

Parameter	ICER in US$	Parameter	ICER in US$
Probability of seeking care from iCCM trained-drug seller (52%)		Probability of seeking care from iCCM trained-CHW (40%)	
40%	144.31	10%	19.86
50%	38.55	20%	21.10
60%	25.43	40%	33.86
70%	20.29	50%	64.81
80%	17.56	60%	Dominated
Drug seller utilization of mRDT (88%)		CHW utilization of mRDT (87%)	
50%	Dominated	50%	21.17
60%	139.52	60%	23.32
70%	62.36	70%	26.14
80%	41.71	80%	30.2
90%	32.13	90%	35.66
100%	26.60	100%	44.62
Prevalence ratio of malaria vs pneumonia vs diarrhea		iCCM cost	
		50% decrease	24.775
		Normal	33.86
0.398 vs 0.63 vs 0.139	28.43	50% increase	42.95
0.624 vs 0.238 vs 0.138	33.86	iCCM effectiveness	
0.662 vs 0.099 vs 0.239	33.69	50% decrease	67.29
Malaria positivity rate (75%)		Normal	33.86
		50% increase	22.53
20%	28.18		
40%	30.14		
60%	32.22		
80%	34.42		
Prevalence of fast breathing (83%)			
40%	41.25		
60%	37.48		
80%	34.34		
100%	31.68		

Scenario analyses were conducted for varying prevalence of malaria, pneumonia and diarrhoea, iCCM cost (50% increase or decrease), and iCCM effectiveness data (50% increase or decrease), and showed that the ICER was robust to changes in most of the parameters except one. A 50% decrease in the effectiveness measure of both interventions resulted in an ICER that was two-fold higher than the central estimate making the iCCM-trained drug seller model less cost-effective.

**Fig. 2:**
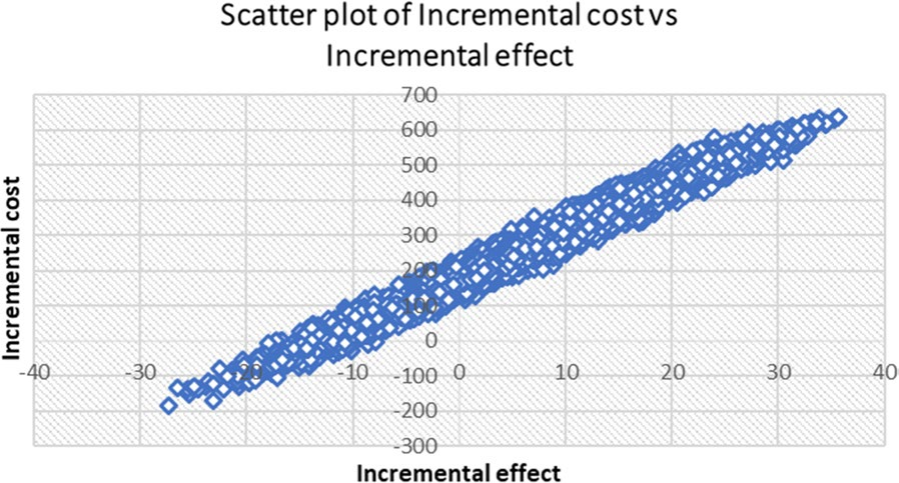
Scatter plot of incremental societal cost in US$ and the incremental number of appropriately treated children resulting from introducing iCCM trained-drug seller, 2018 (US$1 = UGX3727)

**Fig. 3:**
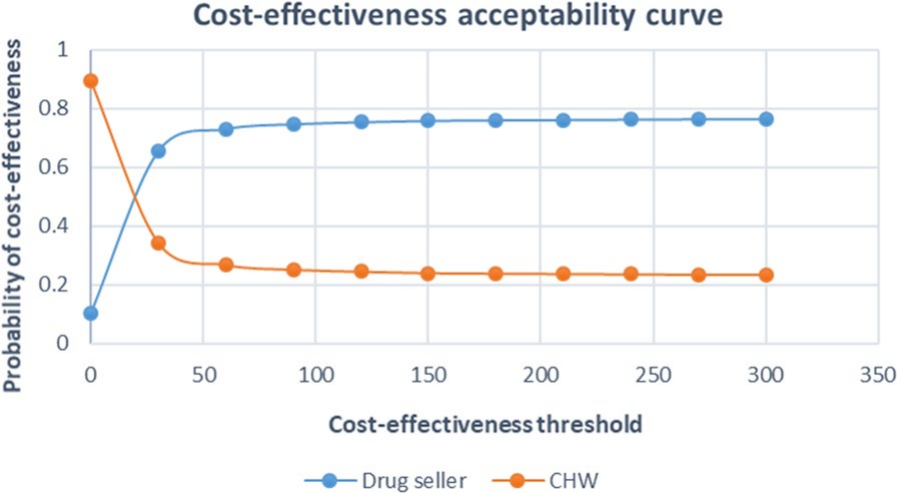
Cost-effectiveness acceptability curve

Results from the probabilistic sensitivity analysis (PSA) are presented in Figs. 2 and 3. Most pairs of incremental costs and effects were situated in the northeastern quadrant of the cost-effectiveness plane, meaning that the change in the number of appropriately treated children and the incremental societal cost was often positive if iCCM-trained drug sellers were used for the treatment of uncomplicated malaria, pneumonia and non-bloody diarrhoea. A few pairs of incremental costs and effects were located in the southwestern and northwestern quadrants of the cost-effectiveness plane, indicating the degree of uncertainty associated with the iCCM trained-drug seller intervention in comparison with the iCCM trained-CHW delivery model (Fig. 2).

According to the cost-effectiveness acceptability curve (CEAC) derived from these pairs of incremental costs and effects (Fig. 3), the probability of the iCCM trained-drug seller delivery model being cost-effective was 40% if a health policy maker’s WTP of about US$15 per appropriately treated child, increasing to 65 and 75% if WTP was US$30 and US$100, respectively. This indicates a high probability that the iCCM trained-drug seller delivery model would be cost-effective from a societal perspective, mainly at a high WTP.

Assuming that the WTP was zero, the probability of iCCM trained-CHW intervention being cost-effective was 90%, decreasing to 30 and 25% if WTP was US$35 and US$100, respectively. At a WTP of US$20, both the iCCM trained-drug seller and CHW intervention have the same probability of cost-effectiveness, which is 50%.

## Discussion

This cost-effective analysis compares the iCCM delivery model utilizing drug sellers against that utilizing CHWs in rural Uganda, basing on the incremental societal cost and effect (in terms of the number of appropriately treated sick U5 children). The health sector costs constituted a more significant part of the total societal cost in both interventions. The iCCM trained-CHW delivery model had a higher health sector cost than the iCCM trained-drug seller model, with the cost of management salaries being the most significant. The iCCM trained-CHW model had more staff involved in its management right from the health facility to the Ministry of Health, leading to a higher cost of management salaries.

CHWs incurred a higher opportunity cost in term of value of time lost than drug sellers since they had to conduct household follow-up visits for U5 children treated and to visit health facilities for quarterly meetings and refresher training, in comparison to the drug seller who never performed any household or health facility visit. However, the total cost incurred was higher in the drug seller group than CHW arm since drug sellers had to buy iCCM drugs from selected wholesalers at a subsidized cost.

U5 caregivers seeking care from drug sellers incurred higher household costs than those seeking care from CHWs. The higher household cost was associated with the enormous direct medical costs (drug cost) and direct non-medical costs (transport to and fro the drug seller and cost of special food for a sick child) that caregivers had to meet when getting care from drug sellers. Additionally, caregivers visiting drug sellers also incurred higher opportunity costs than those visiting CHWs since they had to walk a longer distance to access drug shops [12]. Overall, the total societal cost for diagnosing and treating 100 U5 children was higher in the iCCM trained-drug seller model than the iCCM trained-CHW model: incremental cost being US$292.78.

In terms of the number of appropriately treated U5 cases of malaria, pneumonia and diarrhoea, the iCCM trained-drug seller model resulted in higher appropriately treated U5s than the iCCM trained-CHW model: incremental effect of 9 extra appropriately treated U5 per 100 children. This observation might be due to the low coverage of the iCCM trained-CHW model in addition to the unreliable medicine and equipment supply chains, low motivation, retention of CHWs, weak monitoring, and evaluation systems in some regions of rural Uganda [4, 35].

Using 2018 Ugandan GDP per capita of US$642.78 as the policymakers’ WTP [36], both iCCM trained-drug seller and CHW delivery models are considered cost-effective compared to the do-nothing option (not training drug seller or CHWs on iCCM): average cost-effective ratio for these drug sellers and CHW models are US$19.86 and US$14.13, respectively. This finding is in accord with other studies that demonstrated the cost-effectiveness of the iCCM models utilizing CHWs [23, 37]. However, there are no published studies regarding the cost-effectiveness of the iCCM delivery model utilizing drug sellers. When compared against the iCCM trained-CHW delivery model, the iCCM trained-drug seller model is more cost-effective especially in areas of low malaria prevalence: ICER being US$33.86 per additional appropriately treated U5 case.

One-way sensitivity analysis demonstrated that a low probability of seeking care from drug sellers and low utilization of RDT decrease the cost-effectiveness of the iCCM trained-drug seller delivery model. Low malaria prevalence makes the iCCM trained-drug seller delivery model more cost-effective than high malaria prevalence, as shown in the scenario sensitivity analysis. This observation is because, at low levels of malaria prevalence, there is over-diagnosis of malaria with presumptive treatment, and the use of RDT results in cost savings through avoided ACT prescriptions [38]. Cost-effectiveness can be substantially reduced where drug sellers or CHWs do not adhere to treatment guidelines or do not prescribe according to diagnostic results, where inefficient procurement leads drug sellers or CHWs to run out of drugs or diagnostics frequently, or where quality assurance for drugs or diagnostics is inadequate [38].

According to the CEAC, low WTP results in a higher probability of the iCCM trained-CHW model being cost-effective than the iCCM trained-drug seller model, while at high WTP, the probability of iCCM trained-drug seller being cost-effective is higher.

### Limitations of the study

Most cost [12] and effect data [6, 22] utilized were based on retrospective studies that interviewed U5 caregivers and sometimes drug sellers or CHWs, which thus might have been subjected to recall bias. Data for deriving the distribution of certain parameters like costs and effect were missing; thus, only point estimates were used in PSA.

The authors assumed no co-infection with malaria and pneumonia, malaria and diarrhoea, pneumonia and diarrhoea, or both malaria, pneumonia and diarrhoea. Thus, the possibility of a child presenting with any two or all three illnesses at the same time were not considered in the model.

Better data could improve the model’s accuracy: more effectiveness data for either iCCM trained-drug sellers or CHWs in various rural settings in Uganda based on randomized controlled trials, more data on the cost incurred by drug sellers or CHWs, and household costs in addition to iCCM programme costs.

## Conclusion and recommendations

This study contributed to scarce evidence about the cost-effectiveness of iCCM delivery models utilizing drug sellers and CHWs in rural Uganda. Basing on the average cost-effective ratios, both models were cost-effective compared to the do-nothing option, assuming that the policymaker's WTP was the GDP per capita of Uganda for 2018. Thus, the iCCM trained-drug seller model could complement the iCCM trained-CHW intervention as a strategy to increase access to quality treatment. Therefore, the authors recommend the iCCM trained-drug seller model for particularly low prevalence settings such as Southwestern Uganda and the iCCM trained-CHW model for moderate to high prevalence settings such as Northern Uganda, West Nile, Midwestern Uganda, Eastern Uganda, and Central Uganda.

## Supplementary Information

**Additional file 1: **Probabilities associated with chance nodes of the decision tree analytic model.

## Data Availability

The data that support the findings of this study are available on request from the corresponding author (PL).

## Abbreviations

ACT: Artemisinin-based combination therapy
CEA: Cost-effectiveness analysis
CER: Cost-effectiveness ratio
CHW: Community Health Workers
DT: Dispersible tablets
iCCM: Integrated Community Case Management Intervention for pediatric febrile illness
ICER: Incremental cost-effective ratio
LMIC: Low and middle-income countries
MOH: Uganda Ministry of Health
mRDT: Malaria Rapid Diagnostic Test
MUST: Mbarara University of Science and Technology
NDA: National Drug Authority
ORS: Oral rehydration salts
SA: Sensitivity analysis
SDGs: Sustained development goals
TDR: Special Programme for Research and Training in Tropical Diseases
U5: Under-five
UBOS: Uganda Bureau of Statistics
UGX: Ugandan shillings
UNICEF: United Nations Children’s Fund
USD: United States Dollar
VHT: Village Health Teams
WHO: World Health Organization
