# Supplementary material for: Cost-effectiveness analysis of integrated community case management delivery models utilizing drug sellers and community health workers for treatment of under-five febrile cases of malaria, pneumonia, diarrhoea in rural Uganda
Source: Malar J. 2021 Oct 18;20:407. doi: 10.1186/s12936-021-03944-3 (PMC8524984; doi:10.1186/s12936-021-03944-3)
Supplement: Supplementary file 1 — Additional file 1: Probabilities associated with chance nodes of the decision tree analytic model. [file 12936_2021_3944_MOESM1_ESM.pdf]

Additional file 1. Probabilities associated with chance nodes of the decision tree analytic model.

**Parameter**	**Values**		**Source**
	**iCCM-trained**	**Not iCCM-trained**	
	**Drug sellers**		
Probability of seeking care from iCCM trained- drug sellers vs untrained	0.524	0.476	[39]
Proportion of U5s with malaria vs diarrhea vs pneumonia	0.624 vs 0.138 vs 0.238	0.624 vs 0.138 vs 0.238	[3]
Proportion of U5 with suspected malaria tested with mRDT tested	0.877	0	[39]
Proportion of febrile U5 tested with mRDT that were positive	0.75	0.75	[39]
Proportion of U5s who received ACT given positive mRDT	1	0	[39]
The proportion of U5s who received ACT given negative mRDT	0.091	0	[39]
The proportion of U5s who received ACT given that they were not tested with mRDT	0.213	0.536	[6]
The proportion of U5s diagnosed with diarrhea with loose non-bloody	1	1	[39]
The proportion of U5s who received ORS+Zn given that they had loose non-bloody stools	0.773	0.05	[39]
The proportion of U5s who received ORS+Zn given that they had normal stools	0	0	[39]
The proportion of U5s with suspected pneumonia that had their respiratory rate counted	0.548	0	[39]
Prevalence of fast breathing rate	0.833	0.833	[39]
The proportion of U5s who received amoxicillin given a fast breathing rate	0.96	0	[39]
The proportion of U5s with a normal breathing rate that was given amoxicillin	0	0	[39]
The proportion of U5 who received amoxicillin without respiratory rate being counted	0.260	0.267	[39]

**CHWs**			
Probability of seeking care from iCCM trained CHW vs untrained CHW	0.4	0.6	[12]
Proportion of U5s with malaria vs diarrhea vs pneumonia	0.624 vs 0.138 vs 0.238	0.624 vs 0.138 vs 0.238	[3]
The proportion of U5 with suspected malaria tested with mRDT tested	0.872	0	[40]
The proportion of febrile U5 tested with mRDT that were positive	0.75	0.75	
The proportion of U5s who received ACT given positive mRDT	0.941	0	[41]
The proportion of U5s who received ACT given negative mRDT	0.109	0	[41]
The proportion of U5s who received ACT given that they were not tested with mRDT	0.078	0	[40]
The proportion of U5s diagnosed with diarrhea with loose non-bloody	1	0	[40]
The proportion of U5s who received ORS+Zn given that they had loose non-bloody stools	0.24	0	[12]
The proportion of U5s who received ORS+Zn given that they had normal stools	0	0	
The proportion of U5s with suspected pneumonia that had their respiratory rate counted	0.852	0	[40]
Prevalence of fast breathing rate	0.833	0.883	
The proportion of U5s who received amoxicillin given a fast breathing rate	0.947	0	[41]
The proportion of U5s with a normal breathing rate that was given amoxicillin	0.19	0	[33]
The proportion of U5 who received amoxicillin without respiratory rate being counted	0.078	0	[40]
